# RNA interactions in right ventricular dysfunction induced type II cardiorenal syndrome

**DOI:** 10.18632/aging.202385

**Published:** 2021-01-20

**Authors:** Kaitong Chen, Xiaoxia Huang, Dongxiao Xie, Mengjia Shen, Hairuo Lin, Yingqi Zhu, Siyuan Ma, Cankun Zheng, Lu Chen, Yameng Liu, Wangjun Liao, Jianping Bin, Yulin Liao

**Affiliations:** 1 Department of Cardiology, State Key Laboratory of Organ Failure Research, Nanfang Hospital, Southern Medical University, Guangzhou 510515, China; 2 Department of Oncology, Nanfang Hospital, Southern Medical University, Guangzhou 510515, China; 3 Bioland Laboratory (Guangzhou Regenerative Medicine and Health Guangdong Laboratory), Guangzhou 510005, China

**Keywords:** right ventricular dysfunction, cardiac remodeling, cardiorenal syndrome, whole transcriptome sequencing, co-expression network

## Abstract

Right ventricular (RV) dysfunction induced type II cardiorenal syndrome (CRS) has a high mortality rate, but little attention has been paid to this disease, and its unique molecular characteristics remain unclear. This study aims to investigate the transcriptomic expression profile in this disease and identify key RNA pairs that regulate related molecular signaling networks. We established an RV dysfunction-induced type II CRS mouse model by pulmonary artery constriction (PAC). PAC mice developed severe RV hypertrophy and fibrosis; renal atrophy and dysfunction with elevated creatinine were subsequently observed. Expression profiles in RV and kidney tissues were obtained by whole transcriptome sequencing, revealing a total of 741 and 86 differentially expressed (DE) mRNAs, 159 and 29 DEmiRNAs and 233 and 104 DEcircRNAs between RV and kidney tissue, respectively. Competing endogenous RNA (ceRNA) networks were established. A significant alteration in proliferative, fibrotic and metabolic pathways was found based on GO and KEGG analyses, and the network revealed key ceRNA pairs, such as novel_circ_002631/miR-181a-5p/Creb1 and novel_circ_002631/miR-33-y/Kpan6. These findings indicate that significantly dysregulated pathways in RV dysfunction induced type II CRS include Ras, PI3K/Akt, cGMP-PKG pathways, and thyroid metabolic pathways. These ceRNA pairs can be considered potential targets for the treatment of type II CRS.

## INTRODUCTION

Cardiorenal syndrome (CRS) refers to any disease of the heart and kidneys in which acute or chronic dysfunction of one organ may induce acute or chronic dysfunction of the other [1]. Among the five types of CRS, type II is characterized by chronic heart failure-induced chronic renal dysfunction [2]. Over the past several decades, there have been numerous advances in summarizing the potential mechanisms involved and in finding treatments for type II CRS induced by left ventricular (LV) failure [3, 4]. Although the majority of type II CRS is attributable to LV failure, right ventricular (RV) dysfunction-induced CRS should not be ignored. In fact, there is a growing body of literature recognizes that pulmonary hypertension can induce right heart failure and eventually develop into type II CRS [5–7]. The prevalence of pulmonary hypertension and pulmonary heart disease in patients with end-stage chronic kidney disease is estimated to be approximately 17–56% [8–11], and it has also been reported that pulmonary hypertension is an independent predictor of mortality in end-stage kidney disease [12]. Nonetheless, it remains unclear whether simple right ventricular dysfunction is able to induce renal dysfunction and cause type II CRS.

Among patients with RV dysfunction, acute and chronic kidney diseases are found in 4%–50% and significantly associated with worse outcomes [5, 13]. According to Becirovic-Agic M, et al, after receiving angiotensin II and high salt treatment for 7 days, Balb/CJ mice developed right ventricular dysfunction, followed by fluid retention and peripheral edema [14]. Due to the complicated nature of dysfunction of both the heart and kidneys, treatment of RV dysfunction-induced type II CRS is still limited. Therefore, more effective treatments are needed to slow down or even reverse the development of heart and kidney failure.

To clarify the molecular mechanism of type II CRS caused by right heart dysfunction and find new therapeutic targets, we sought to examine the molecular alterations of RV dysfunction-induced type II CRS. In recent years, the therapeutic functions of noncoding RNAs have attracted much attention, and in this study, we focused on expression profiles of mRNAs, microRNAs (miRNAs) and circular RNAs (circRNAs).

With the help of whole transcriptome RNA sequencing technology, the overall transcriptional activity in any species can be detected at the single nucleotide level, and corresponding expression profile information can be obtained. MiRNAs and circRNAs are considered to be closely related to the development of cardiovascular or kidney diseases. The former are endogenous noncoding RNA molecules consisting of approximately 22 nucleotides that have recently been reported to be both potential protective targets of type IV CRS [15, 16] and linked to the development of cardiovascular or kidney diseases [17–19]. Indeed, miRNAs regulate pathogenesis of the heart and kidney in other conditions by functioning as competing endogenous RNAs (ceRNAs) of circRNAs and mediating downstream mRNAs [20–22].

In this study, we developed an RV dysfunction-induced type II CRS mouse model by pulmonary artery constriction (PAC). Through bioinformatics analysis of heart and kidney transcriptomes in sham and PAC mice, we aimed to investigate circRNA, miRNA and mRNA transcriptome expression profiles and to identify potential therapeutic targets for regulating molecular signaling networks in RV dysfunction-induced type II CRS.

## RESULTS

### PAC induced RV dysfunction

**Figure 1:**
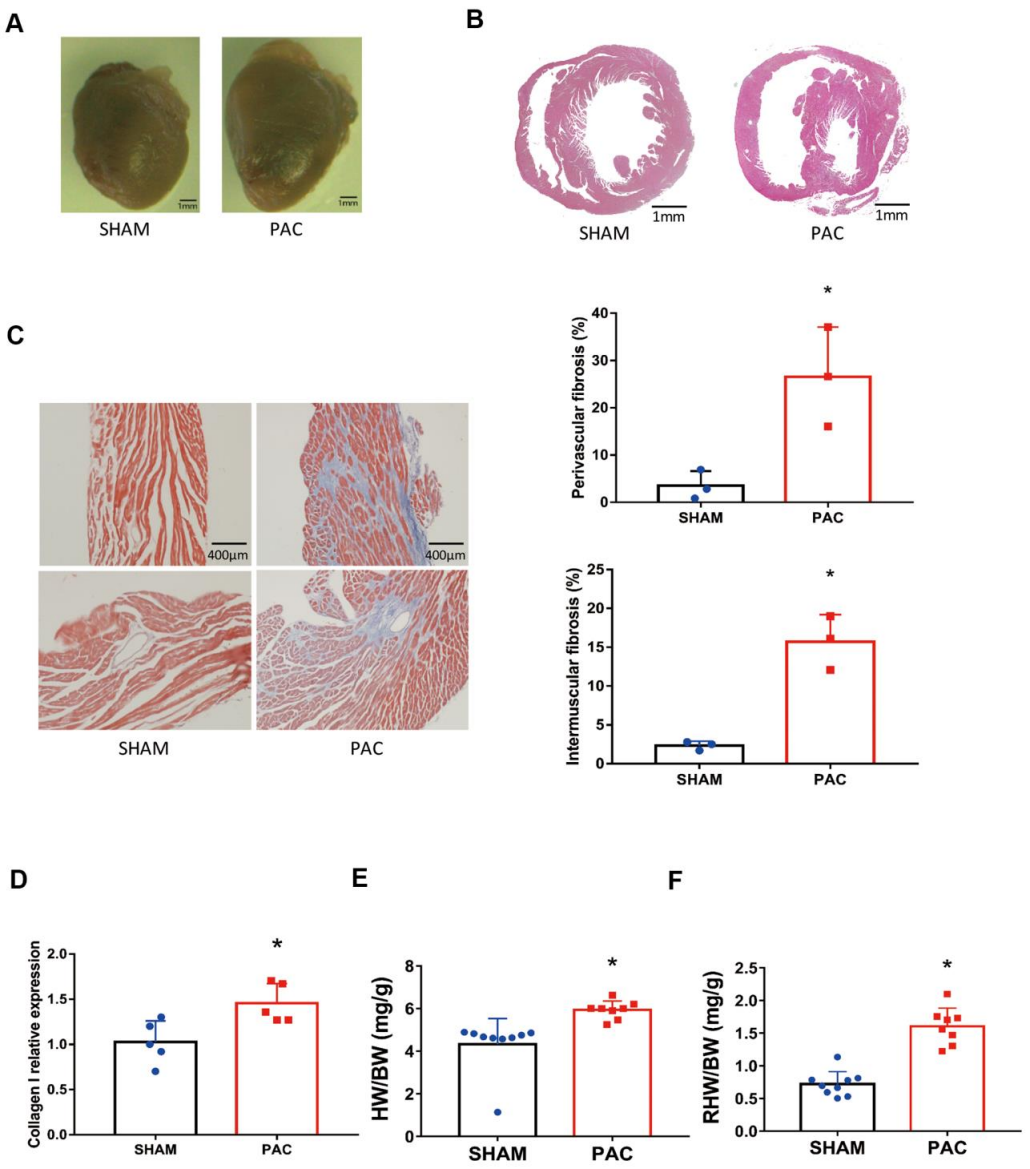
**Pulmonary artery constriction induced right ventricular (RV) morphology alterations.** (**A**) Representative right ventricular hypertrophy at 4 weeks after pulmonary artery constriction (PAC). (**B**) Hematoxylin and eosin (H&E) staining of RV tissue. (**C**) Azan-Masson staining of the RV in intermuscular and perivascular parts and their corresponding fibrotic area percentage. (**D**) Gene expression level of collagen I. (**E**) The heart weight to body weight ratio (HW/BW). (**F**) The right heart weight to body weight ratio (RHW/BW). *P < 0.05 vs the corresponding sham group; Scale bar=1 mm for panels **A** and **B**, scale bar = 400 μm for panel **C**.

Compared with the sham groups four weeks after surgery, the PAC group showed an enlargement of the right ventricle (Figure 1A, 1B). Masson staining revealed an increase in cardiac fibrosis in both the perivascular and intermuscular areas (Figure 1C), and expression of the fibrotic marker collagen I increased significantly (Figure 1D). Similarly, heart weight (Figure 1E) and right heart weight (Figure 1F) normalized to body weight increased significantly after PAC surgery.

**Figure 2:**
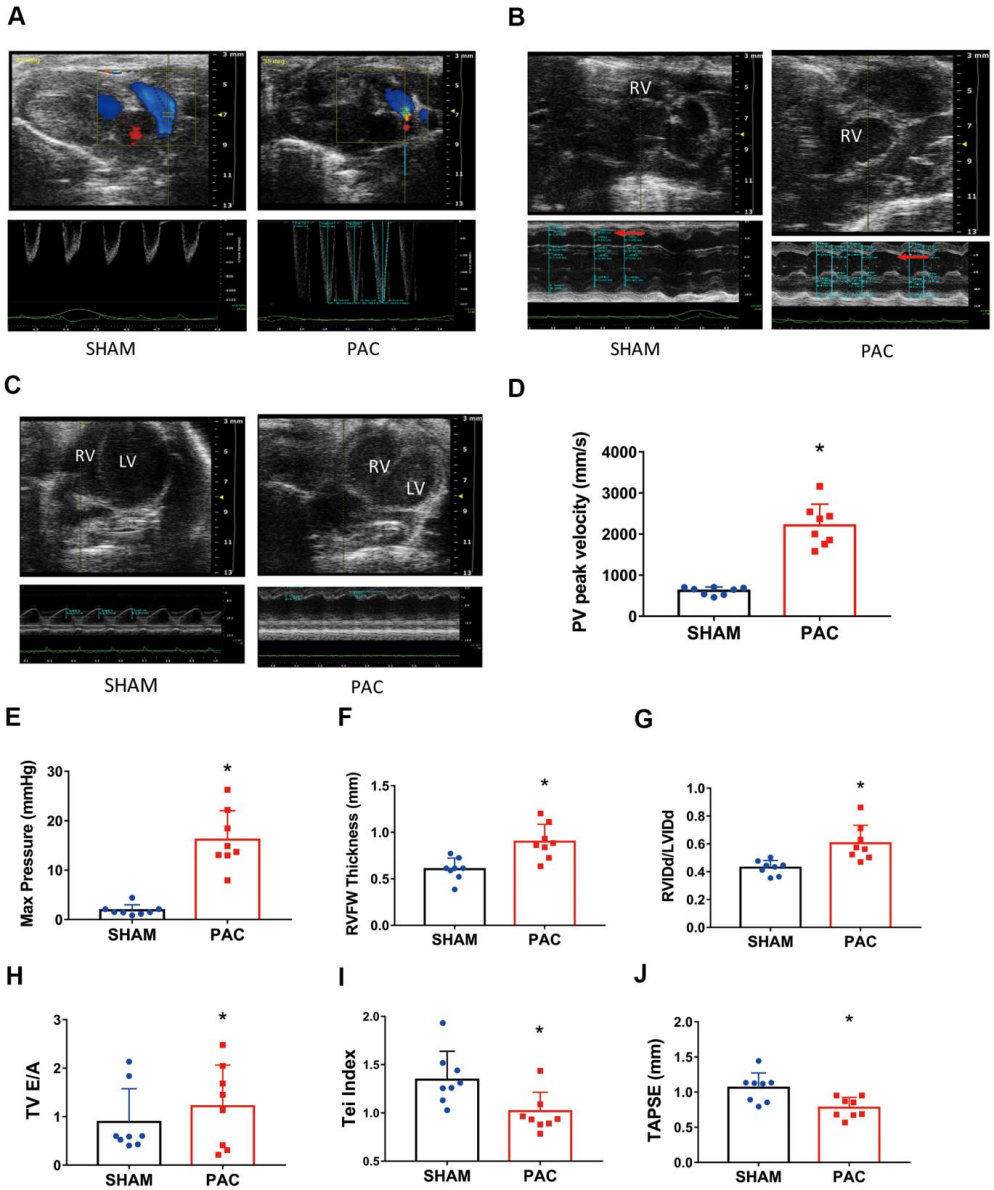
**Pulmonary artery constriction induced right ventricular (RV) dysfunction.** (**A**–**C**) Representative recordings of M-mode echocardiographic images; the red arrows point to the RV chamber. (**D**) The pulmonary valve (PV) peak velocity. (**E**) The pulmonary valve (PV) peak pressure. (**F**) The RV free wall thickness. (**G**) The ratio of right ventricular diastolic internal diameter to left ventricular diastolic internal diameter (RVIDd/LVIDd). (**H**) The tricuspid valve (TV) E/A ratio. (**I**) The RV myocardial performance index (or Tei index). (**J**) Tricuspid annular plane systolic excursion (TAPSE) in the sham and PAC groups. PAC: Pulmonary artery constriction. *P < 0.05 vs the corresponding sham group.

Echocardiography revealed higher PV peak velocity (Figure 2A, 2D), higher PV max pressure (Figure 2E), thicker RV free wall (Figure 2B, 2F), and larger RV internal diameter (Figure 2C, 2G) in the PAC group than in the corresponding sham group. The significant differences in the tricuspid valve E/A ratio (TV E/A) and Tei index indicated RV diastolic dysfunction in the PAC group (Figure 2H, 2I). Moreover, a significantly lower tricuspid annular plane systolic excursion (TAPSE) in the PAC group indicated the existence of systolic dysfunction (Figure 2J). Taken together, PAC successfully led to RV dysfunction.

### RV dysfunction induced renal dysfunction

**Figure 3:**
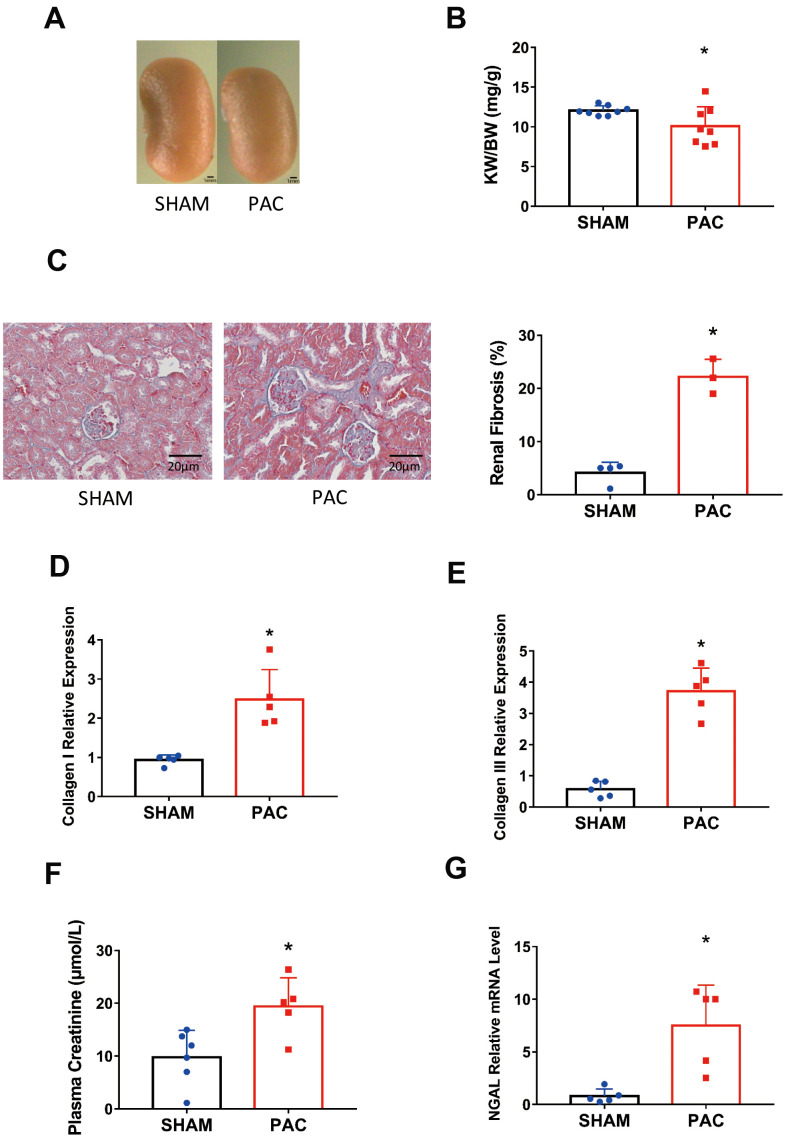
**Pulmonary artery constriction induced renal dysfunction.** (**A**) Representative pictures of the kidney in the sham and PAC groups. (**B**) The kidney weight to body weight ratio (KW/BW). (**C**) HE staining of the section around the glomerulus and their corresponding fibrosis area percentage. (**D**, **E**) Gene expression level of collagen I and collagen III in the kidney. (**F**) Plasma creatinine concentration in the two groups. (**G**) Gene expression level of neutrophil gelatinase-associated lipocalin (NGAL). PAC: Pulmonary artery constriction. *P < 0.05 vs the corresponding sham group. Scale bar=1 mm for panel A and scale bar = 20 μm for panel **C**.

In addition to RV dysfunction, we found that PAC mice showed signs of renal dysfunction. PAC mice displayed obvious renal atrophy with statistical significance (Figure 3A, 3B). Renal fibrosis in the PAC group was detected by Masson staining with an increase of approximately 15% of the fibrotic area compared to the sham group (Figure 3C). The fibrotic markers collagen I and III increased simultaneously (Figure 3D, 3E). We also examined renal function, noting a significant increase in plasma creatinine in the PAC group compared with the sham group (Figure 3F). Moreover, the level of neutrophil gelatinase-associated lipocalin (NGAL), an indicator of kidney injury [23], was significantly higher in the PAC group (Figure 3G). Thus, we generated an RV dysfunction-induced type II CRS mouse model.

### Changes in coding and noncoding RNA expression patterns in response to PAC

Research to date has rarely addressed coding and noncoding RNA expression profiles in RV dysfunction-induced type II CRS models. To obtain a comprehensive understanding of transcriptome alterations in the heart and kidney, we performed whole transcriptome RNA sequencing on these tissues in sham and PAC mice.

A total of 741 DEmRNAs (556 upregulated and 185 downregulated) were detected in RV tissue and 86 DEmRNAs (49 upregulated and 37 downregulated) in kidney tissue according to the threshold of FDR < 0.05 and | log2(Fold Change) | ≥ 1. The top ten up- and downregulated DEmRNAs in the RV and kidney are shown in Tables 1, 2, respectively. Hierarchically clustered heat maps and volcano plots illustrated DEmRNAs in different tissues between the PAC and sham groups (Supplementary Figure 1A, 1B).

**Table 1:** Top ten up- and down-regulated mRNAs in the RV tissue between sham and PAC groups.

**ID**	**Log2 (Fold Change)**	**p-value**	**FDR**
*Gdf15*	11.20823	2.28E-10	1.53E-07
*Plxnb2*	10.31515	1.31E-06	0.000341
*Rftn1*	10.02237	2.58E-05	0.0037
*Tnfrsf26*	9.554589	6.04E-06	0.001144
*P4ha3*	9.430453	4.33E-07	0.000131
*Senp3*	9.344296	0.000822	0.048977
*Syvn1*	9.23362	4.21E-05	0.005276
*Erlin1*	9.113742	0.000349	0.025814
*Prss16*	9.050529	7.61E-08	2.97E-05
*Pcnt*	8.906891	1.41E-07	5.19E-05
*Myom1*	-10.6653	6.75E-05	0.007798
*Abcc9*	-10.6582	3.70E-05	0.004816
*Sptan1*	-10.5527	2.27E-05	0.00332
*Ctnnbl1*	-9.52356	0.00045	0.031368
*Ciz1*	-9.49586	0.000274	0.022008
*Anks1*	-9.43045	1.31E-07	4.85E-05
*Ctnnd1*	-9.08746	0.000196	0.017636
*Shf*	-8.98299	0.000752	0.046107
*Ank1*	-8.76818	2.08E-06	0.000492
*Ccdc88c*	-8.29462	2.68E-07	8.70E-05

FDR: False Discovery Rate.

**Table 2:** Top ten up- and down-regulated mRNAs in the kidney tissue between sham and PAC groups.

**ID**	**Log2 (Fold Change)**	**p-value**	**FDR**
*Puf60*	13.05155	4.32E-12	4.76E-08
*Cd151*	12.65195	2.73E-08	0.000116
*Rpl22*	12.18488	1.75E-05	0.017925
*Krcc1*	11.44501	7.26E-06	0.009098
*Eif4g1*	11.43359	6.05E-13	1.11E-08
*Aco1*	11.42206	2.09E-05	0.020577
*Aldh6a1*	11.4168	2.85E-05	0.024937
*Hykk*	10.98157	1.22E-05	0.013683
*Ifit2*	10.84235	7.35E-05	0.048284
*Scp2*	10.64776	2.21E-12	3.04E-08
*Gsr*	-13.0458	1.34E-06	0.002742
*Ggt1*	-12.8134	2.09E-06	0.003843
*Nr1h3*	-12.4767	7.10E-05	0.047419
*Zfp961*	-11.7117	2.54E-10	2.16E-06
*Igf2r*	-11.6375	3.47E-08	0.000119
*Rragc*	-11.5108	2.98E-09	1.83E-05
*Anxa2*	-11.2842	3.41E-05	0.028089
*Fcho2*	-11.043	3.13E-05	0.0266
*Ndufs1*	-10.9787	8.11E-06	0.009943
*P4ha2*	-10.5831	3.51E-05	0.028495

PAC: Pulmonary Artery Constriction, FDR: False Discovery Rate.

In addition, 159 DEmiRNAs were found in the RV, of which 123 were upregulated and 36 downregulated. In the kidney, there were 29 DEmiRNAs including 25 that were upregulated and four that were downregulated. The top ten up- and downregulated DEmiRNAs in the RV are shown in Table 3, and the top ten up- and six downregulated DEmiRNAs in the kidney are shown in Table 4. Visualization DEmiRNAs in different tissues between PAC and sham groups using hierarchical clustering heat maps and volcano plots is shown in Supplementary Figure 1C, 1D.

**Table 3:** Top ten up- and down-regulated miRNAs in the RV tissue between sham and PAC groups.

**ID**	**Log2 (Fold Change)**	**p-value**
miR-205-x	6.886257	4.42E-06
miR-744-y	5.190141	0.000567
novel-m0041-5p	5.181977	2.85E-05
mmu-miR-344b-3p	4.890155	0.002496
miR-671-x	4.730314	0.004923
miR-33-x	4.630289	0.024382
miR-455-x	4.603715	0.020292
mmu-miR-205-5p	4.47903	1.58E-12
mmu-miR-208b-3p	4.374356	2.08E-31
miR-132-x	4.346531	0.010407
miR-10018-x	-5.19582	0.014163
novel-m0060-5p	-4.76235	0.000202
miR-3959-x	-4.74093	0.029248
mmu-miR-215-3p	-4.38943	0.020228
miR-154-y	-3.98571	0.031775
miR-3958-y	-3.98571	0.03155
mmu-miR-1958	-3.8955	0.020479
miR-507-y	-3.24738	0.007582
mmu-miR-202-5p	-3.20089	0.011996
miR-10390-x	-2.37447	4.84E-08

RV: Right Ventricular, PAC: Pulmonary Artery Constriction.

**Table 4:** Top ten up- and top six down-regulated miRNAs in the kidney tissue between sham and PAC groups.

**ID**	**Log2 (Fold Change)**	**p-value**
miR-276-y	5.810186	0.009873
miR-13-y	5.654034	0.011793
bantam-y	5.089244	0.018082
miR-33-y	4.660723	0.000654
miR-1338-x	4.652142	0.03376
miR-1388-x	4.652142	0.033981
miR-380-y	4.611645	0.034829
mmu-miR-7668-5p	4.293223	0.003289
mmu-miR-7667-3p	4.274411	0.008729
mmu-miR-202-5p	4.132496	0.002331
mmu-miR-541-3p	-4.33099	0.005716
mmu-miR-6986-5p	-2.81436	0.038717
mmu-miR-215-5p	-1.44345	0.000349
mmu-miR-138-5p	-0.79273	0.044968
mmu-miR-383-5p	-0.49446	0.035023
mmu-miR-1943-5p	-0.48098	0.041138

PAC: Pulmonary Artery Constriction.

Based on mapping reads from filtered ribosomes to the reference genome, 13,648 circRNAs were identified, with 467 existing and 13,182 novel ones. In the RV, there were 130 upregulated circRNAs and 102 downregulated circRNAs, of which 59.27% were exonic, 16.33% were antisense, 7.35% were intronic and 1.6% were intergenic (Supplementary Figure 2A, 2B). A total of 41 and 62 DEcircRNAs in the kidney were detected, with exonic, antisense, intronic and intergenic circRNAs accounting for 66.44%, 10.18%, 6.28% and 1.65%, respectively (Supplementary Figure 2C, 2D). The top ten up- and downregulated DEcircRNAs in RV and kidney are provided in Tables 5, 6, respectively. These circRNAs are widely distributed among all chromosomes, including the mitochondrial chromosome and the sex chromosomes. Among all circRNAs identified, the top three chromosomes of circRNA distribution were found to be chromosomes 2 (chr 2), chr 1 and chr 11 in the sham and PAC groups for both the RV and kidney (Supplementary Figure 2E). The identified circRNAs are mainly between 201 to 700 bases in length (Supplementary Figure 2F).

**Table 5:** Top ten up- and down-regulated circRNAs in the RV tissue between sham and PAC groups.

**ID**	**Log2(FC)**	**P-value**	**Source Gene**	**Annotation Type**
novel_circ_004124	18.11539	0.001302	NA	intergenic
novel_circ_004845	18.00805	0.000433	ENSMUSG00000022906	one_exon
novel_circ_012103	17.95287	0.000225	ENSMUSG00000041308	annot_exons
novel_circ_010930	17.94796	0.034129	ENSMUSG00000030287	annot_exons
novel_circ_011235	17.94164	0.000932	ENSMUSG00000030562	annot_exons
novel_circ_012417	17.74917	0.005109	ENSMUSG00000032267	annot_exons
novel_circ_011834	17.73501	0.001967	ENSMUSG00000031644	annot_exons
novel_circ_007624	17.73014	0.001617	ENSMUSG00000033396	annot_exons
novel_circ_000124	17.70371	0.00173	ENSMUSG00000026134	annot_exons
novel_circ_010110	17.67082	0.021793	ENSMUSG00000063146	annot_exons
novel_circ_000513	-18.2015	0.0128	ENSMUSG00000026319	annot_exons
novel_circ_004699	-18.1722	0.000869	ENSMUSG00000022788	annot_exons
novel_circ_004025	-18.0592	0.000279	ENSMUSG00000033083	annot_exons
novel_circ_003066	-17.8837	0.017077	ENSMUSG00000021313	annot_exons
novel_circ_013251	-17.6135	0.000323	ENSMUSG00000064351	antisense
novel_circ_000286	-17.5883	0.000922	NA	intergenic
novel_circ_000034	-17.535	0.023839	ENSMUSG00000048960	annot_exons
novel_circ_006576	-17.519	0.025731	ENSMUSG00000039652	exon_intron
novel_circ_012489	-17.5157	0.014054	ENSMUSG00000032340	annot_exons
novel_circ_005069	-17.4659	0.008808	ENSMUSG00000069729	annot_exons

RV: Right Ventricular, PAC: Pulmonary Artery Constriction.

**Table 6:** Top ten up- and down-regulated circRNAs in the RV tissue between sham and PAC groups.

**ID**	**Log2(FC)**	**P-value**	**Source Gene**	**Annotation Type**
novel_circ_006516	17.97984	0.002568	ENSMUSG00000024887	annot_exons
novel_circ_002315	17.88867	0.002487	ENSMUSG00000018412	annot_exons
novel_circ_011094	17.86901	0.012238	ENSMUSG00000006599	annot_exons
novel_circ_005441	17.74596	0.013776	ENSMUSG00000024064	annot_exons
novel_circ_006835	17.7362	0.019706	ENSMUSG00000026726	annot_exons
novel_circ_003137	17.66716	0.012486	ENSMUSG00000006191	annot_exons
novel_circ_003258	17.65575	0.026084	ENSMUSG00000021488	annot_exons
novel_circ_011050	17.59386	0.048764	ENSMUSG00000001249	annot_exons
novel_circ_007417	17.58647	0.040513	ENSMUSG00000044033	exon_intron
novel_circ_009319	17.57595	0.027606	ENSMUSG00000020220	annot_exons
novel_circ_000901	-18.0099	0.027084	ENSMUSG00000026604	one_exon
novel_circ_003938	-17.8339	0.001138	ENSMUSG00000033487	annot_exons
novel_circ_010707	-17.7456	0.023897	ENSMUSG00000000441	annot_exons
novel_circ_006640	-17.6834	0.026371	ENSMUSG00000025176	annot_exons
novel_circ_011053	-17.6793	0.0105	ENSMUSG00000066571	one_exon
novel_circ_000908	-17.6505	0.022348	ENSMUSG00000089872	exon_intron
novel_circ_003196	-17.6372	0.025622	ENSMUSG00000038518	annot_exons
novel_circ_002047	-17.5872	0.040051	ENSMUSG00000038178	annot_exons
novel_circ_011132	-17.5706	0.033965	ENSMUSG00000030513	annot_exons
novel_circ_009496	-17.5641	0.01143	ENSMUSG00000028986	annot_exons

RV: Right Ventricular, PAC: Pulmonary Artery Constriction.

### Validation of RNA sequencing data by RT-PCR

**Figure 4:**
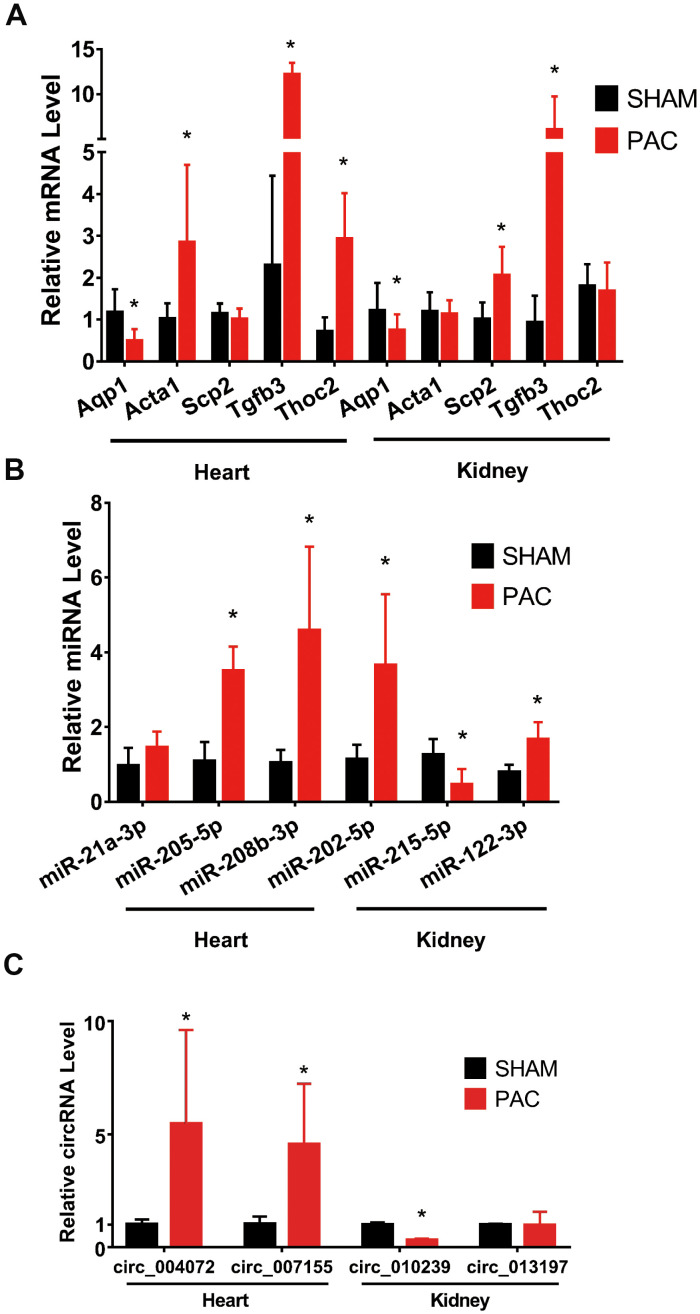
**Validation of mRNAs and noncoding RNAs in the right ventricle and kidney.** (**A**) qRT-PCR validation of selected mRNAs between the sham and PAC groups. (**B**) qRT-PCR validation of selected miRNAs between the sham and PAC groups. (**C**) Validation of circRNAs between the sham and PAC groups; n = 5 in each group. *P < 0.05 vs the corresponding sham group. PAC: pulmonary artery constriction.

To validate the sequencing results, we randomly selected five mRNAs, three miRNAs and two circRNAs in the two tissues for qRT-PCR. Aqp1 was downregulated in both tissues. By contrast, Acta1 was significantly upregulated in the RV but remained the same in the kidney. Although expression of Scp2 in the RV was not significantly different between the sham and PAC groups, it was significantly increased in the kidney. Thoc2 was only increased in the RV between the PAC and sham groups, but there was no significant change in the kidney. Supporting the existence of fibrosis in the RV and kidney, Tgfβ3 expression in both tissues was significantly higher in PAC mice than in sham mice (Figure 4A).

Regarding miRNA expression levels, miR-205-5p and miR-208b-3p were significantly upregulated in the RV tissue in the PAC group compared with the sham group; miR-21a-3p displayed an increasing trend with no significant difference between the two groups. MiR-202-5p and miR-122-3p were significantly upregulated in the kidneys of PAC mice compared with sham mice, whereas miR-215-5p was significantly downregulated (Figure 4B).

The qRT-PCR results showed mm9_circ_004072 and mm9_circ_007155 to be significantly upregulated in the RV of PAC mice. Mm9_circ_010239 showed a downregulation of approximately 3 times in the kidney, though expression of mm9_circ_013197 remained the same (Figure 4C).

All of the findings were consistent with the sequencing results, potently validating the reliability of our RNA sequencing.

### Functional prediction of DEmiRNAs in RV tissue

To understand the potential molecular mechanisms of RV dysfunction, we first constructed pairs of miRNAs and their target genes with a negative correlation using a threshold of *P* < 0.05 and | log2(Fold Change) | ≥ 1. Functional analysis of the negatively regulated pairs was conducted according to the regulation direction of the target mRNAs (upregulated mRNAs with downregulated miRNAs group; downregulated mRNAs with upregulated miRNAs group).

A total of 2227 pairs containing 43 miRNAs and 210 target mRNAs were found in RV tissue. GO analysis of these upregulated mRNAs in the RV of PAC mice revealed significant enrichment in the biological process of positive regulation of the cell cycle and Ras protein signal transduction (Supplementary Figure 3A); and the downregulated mRNAs in RV showed enrichment mainly in metabolic processes, including low-density lipoprotein receptor particle metabolic process, carboxylic acid biosynthetic process and organic acid biosynthetic process (Supplementary Figure 3B). Based on KEGG analysis, the upregulated mRNAs in PAC mice were mostly enriched in the PI3K-Akt signaling pathway and focal adhesion pathway (Supplementary Figure 3C), whereas the downregulated mRNAs were mostly enriched in the PPAR signaling pathway (Supplementary Figure 3D).

### Functional prediction in renal tissue

Renal dysfunction was observed in the PAC group, in order to uncover genetic alterations related to kidney dysfunction, GO and KEGG analyses were performed. As depicted in Supplementary Figure 1D, we noticed an abnormal opposite expression pattern of the first sample in the PAC group. To eliminate bias, the data of this sample were not included in the analysis.

A total of 140 miRNA-mRNA pairs consisting of 46 DEmRNAs and 31 DEmiRNAs were involved in the analysis. GO analysis of the upregulated mRNAs in renal tissue of the PAC group revealed significant biological process enrichment in positive regulation of cell migration, small GTPase-mediated signal transduction and positive regulation of neuron differentiation (Supplementary Figure 4A). and the downregulated mRNAs in the kidneys were also enriched mainly in metabolic processes including cofactor, coenzyme and lipid metabolic processes (Supplementary Figure 4B). KEGG analysis of upregulated mRNAs indicated enrichment in the PI3K-Akt signaling pathway, which was interestingly the same as for RV tissue (Supplementary Figure 4C), and the downregulated mRNAs were enriched in the phospholipase D signaling pathway and apelin signaling pathway (Supplementary Figure 4D).

### Construction of regulation networks of miRNA-mRNA

To demonstrate the dysregulated relationship of miRNA-mRNA pairs, we first built miRNA-mRNA regulation network based on the expression levels of DEmRNAs and DEmiRNAs. In the RV tissue sample, to highlight the contribution of hub miRNA-mRNA pairs to the fibrotic and metabolic regulation, we selected DE pairs with the threshold of the abstract value of log_2_(Fold Change) ≥ 10, with 34 DEmRNAs and 132 DEmiRNAs consisting of 333 pairs involved in the network (Supplementary Figure 5A). In the renal tissue sample, 46 DEmRNAs and 31 DEmiRNAs were involved in the network. The top three upregulated DEmiRNAs were miR-33-y, miR-7667-3p and miR-7668-5p. The top three downregulated DEmiRNAs were miR-6986-5p, miR-541-3p and miR-671-x (Supplementary Figure 5B).

### Construction of ceRNA networks of circRNA-miRNA-mRNA

Then, based on the circRNA-miRNA and miRNA-mRNA targeting relationship, we predicted ceRNA pairs of circRNA-miRNA-mRNA. Interestingly, functional analysis of the ceRNA network showed similar to those of miRNA-mRNA: the most significantly changed biological processes were fibrosis, cell proliferation, and metabolism-related processes.

**Figure 5:**
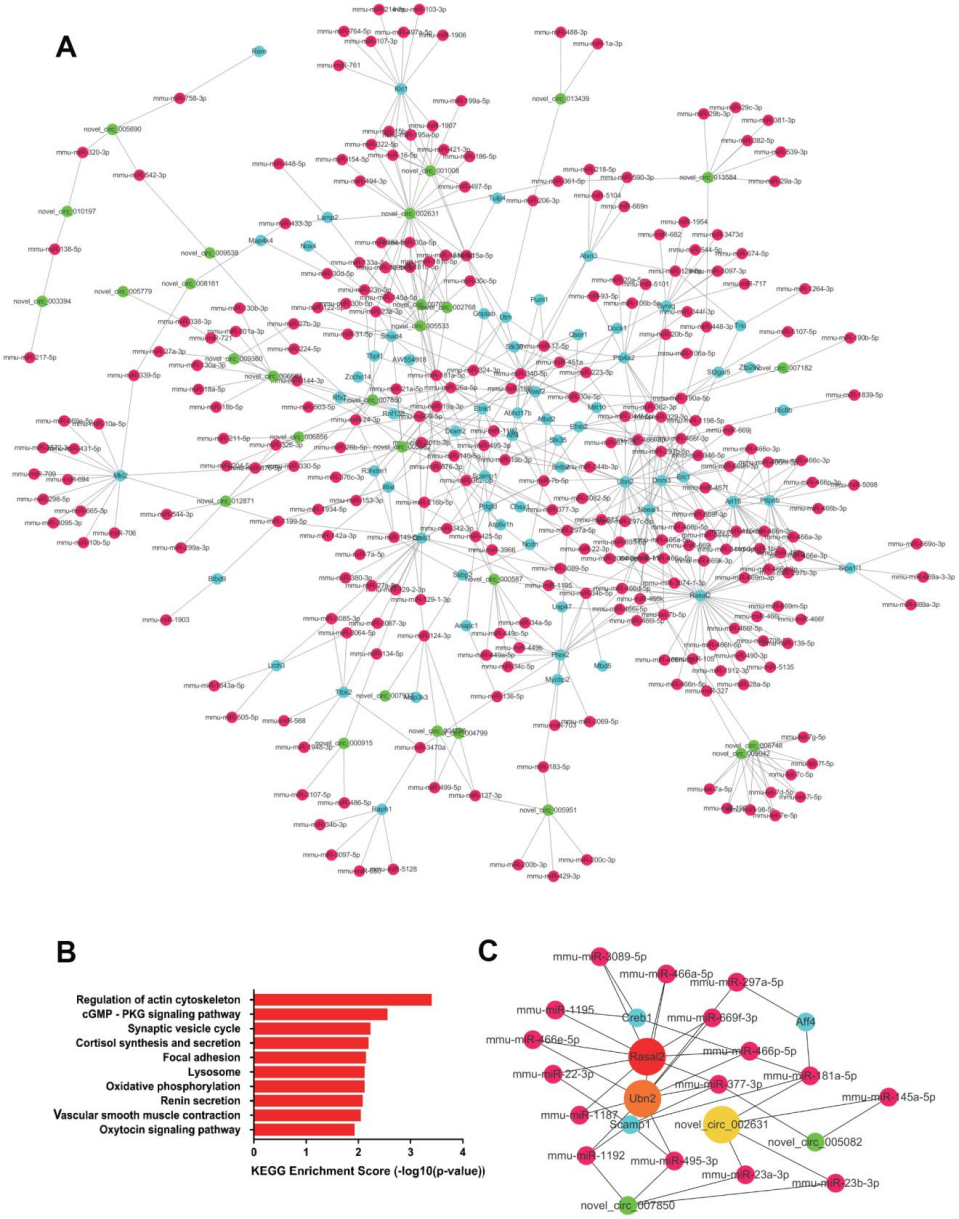
**The upregulated ceRNA network in RV tissue.** (**A**) Paired upregulated mRNA and corresponding circRNA and miRNA; the CeRNA network is composed of circRNA, miRNA and mRNA in RV tissue. The green node represents circRNA, the red node represents miRNA, and the blue node represents mRNA. (**B**) KEGG analysis of ceRNA pairing. (**C**) Screened network of key RNA nodes. Key nodes are represented by larger circles; the darker is the color, the greater is the number of related nodes.

**Figure 6:**
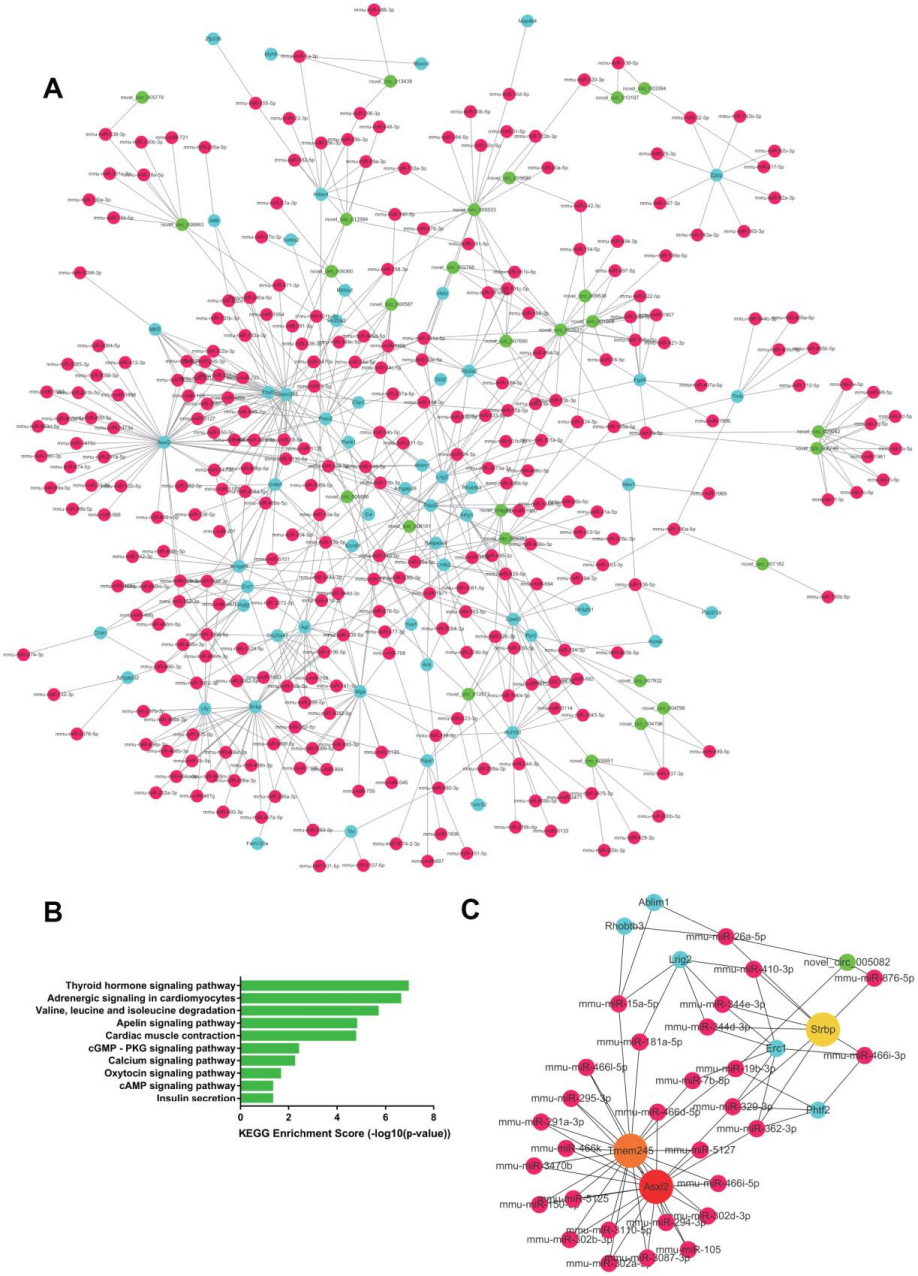
**The downregulated ceRNA network in RV tissue.** (**A**) Paired downregulated mRNA and corresponding circRNA and miRNA; the CeRNA network is composed of circRNA, miRNA and mRNA in RV tissue. The green node represents circRNA, the red node represents miRNA, and the blue node represents mRNA. (**B**) KEGG analysis of ceRNA pairing. (**C**) Screened network of key RNA nodes. Key nodes are represented by larger circles; the darker is the color, the greater is the number of related nodes.

A total of 1104 upregulated ceRNA pairs were found in the RV tissue, including 32 circRNAs, 263 miRNAs, and 66 mRNAs. While 1,236 ceRNA pairs were downregulated, including 32 circRNAs, 295 miRNAs, and 58 target mRNAs. The network of the paired downregulated ceRNA is shown in Figures 5A, 6A. In right heart tissue, cardiac hypertrophy and metabolic processes were both dysregulated. The significantly upregulated ceRNA pairs were mainly enriched in the actin cytoskeleton regulatory pathway and the cGMP-PKG signaling pathway (Figure 5B). Next, according to the number of edges connected with each node, we selected hub RNAs located in key positions in the network (Figure 5C). In the upregulated ceRNA network for the RV, the RNAs with the highest number of edges were Rasal2, Ubn2 and novel_circ_002631. As illustrated in Figure 6B, the significantly downregulated ceRNA pairs were mainly enriched in metabolic pathways, including the thyroid hormone signaling pathway, adrenergic signaling pathway, valine, leucine and isoleucine degradation. Hub RNAs were Asxl2, Tmem245 and Strbp (Figure 6C).

**Figure 7:**
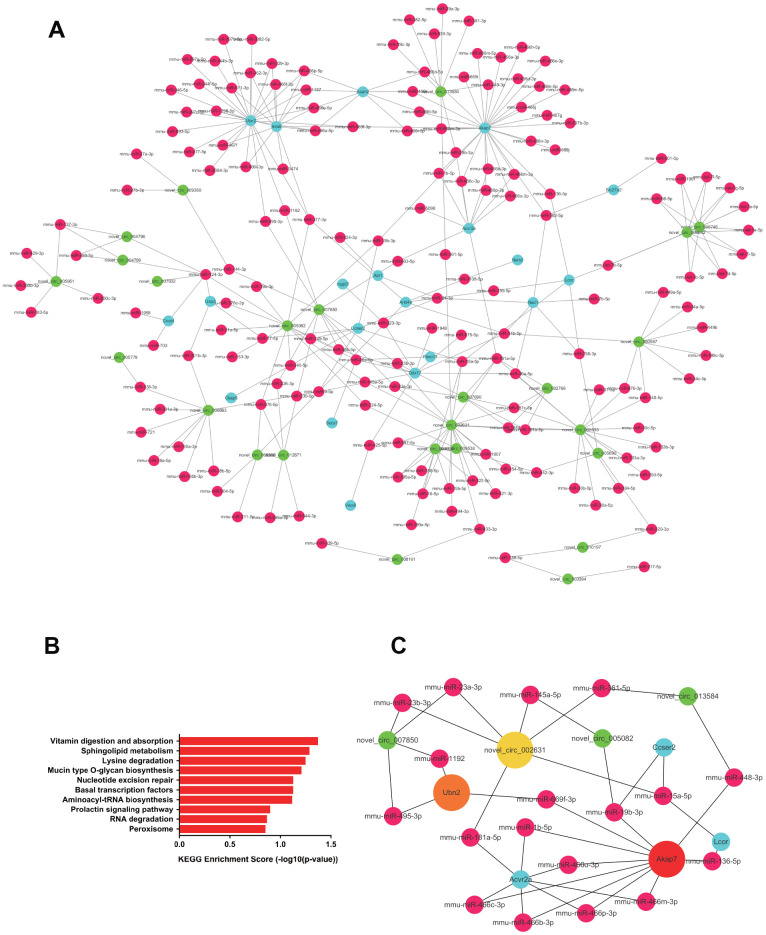
**The upregulated ceRNA network in renal tissue.** (**A**) Paired upregulated mRNA and corresponding circRNA and miRNA; the CeRNA network is composed of circRNA, miRNA and mRNA in renal tissue. The green node represents circRNA, the red node represents miRNA, and the blue node represents mRNA. (**B**) KEGG analysis of ceRNA pairing. (**C**) Screened network of key RNA nodes. Key nodes are represented by larger circles; the darker is the color, the greater is the number of related nodes.

**Figure 8:**
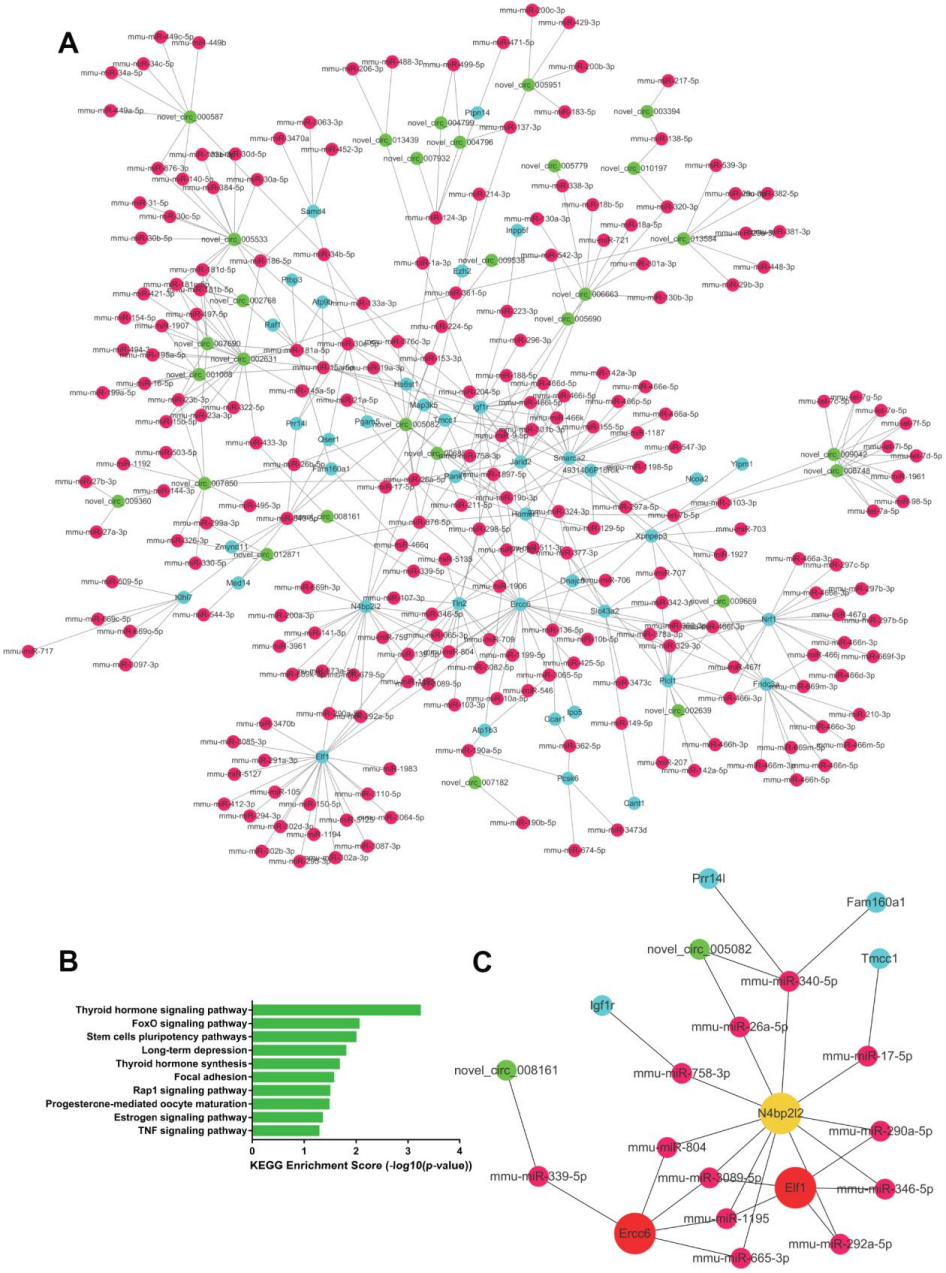
**The downregulated ceRNA network in renal tissue.** (**A**) Paired downregulated mRNA and corresponding circRNA and miRNA; the CeRNA network is composed of circRNA, miRNA and mRNA in renal tissue. The green node represents circRNA, the red node represents miRNA, and the blue node represents mRNA. (**B**) KEGG analysis of ceRNA pairing. (**C**) Screened network of key RNA nodes. Key nodes are represented by larger circles; the darker is the color, the greater is the number of related nodes.

In kidney tissue, 608 upregulated ceRNA pairs were selected, including 32 circRNAs, 175 miRNAs, and 20 mRNAs (Figure 7A). GO analysis suggested metabolism to be the most significantly dysregulated biological process, whereas KEGG analysis showed the significantly upregulated pairs to be enriched in pathways such as vitamin metabolism, sphingolipid metabolism and lysine degradation (Figure 7B). Hub RNAs were Akap7, Ubn2 and novel_circ_002631 (Figure 7C). A total of 834 ceRNA pairs were downregulated, as shown in Figure 8A, consisting of 32 circRNAs, 230 miRNAs and 41 mRNAs. Downregulated pairs were most enriched in the thyroid hormone signaling pathway and the FoxO signaling pathway (Figure 8B), with Elf1, Ercc6 and N4bp2l2 as hub RNAs (Figure 8C).

## DISCUSSION

According to the latest guidelines, CRS can be divided into five types [24]. Type II CRS is estimated to exist in 25% to 63% of patients with heart failure [25]. However, the pathophysiology and molecular mechanisms of renal dysfunction in the setting of RV dysfunction are largely unknown. To determine whether RV dysfunction contributes to renal dysfunction, we established a mouse model using PAC to induce RV dysfunction, which was confirmed by echocardiography and histological examinations. As expected, we found that PAC mice developed renal dysfunction, as we detected increases in the plasma creatinine concentration, NGAL expression and renal fibrosis. We then performed RNA sequencing to clarify the potential molecular alterations involved.

CircRNAs and miRNAs can repress protein translation by binding to and competitively regulating target mRNAs. In this study, the landscape of circRNAs, miRNAs and mRNAs in RV and renal tissue of the PAC and sham groups was obtained by high-throughput sequencing and bioinformatics analysis. Hundreds of DERNAs were detected in both heart and kidney tissues. Among them, the expression levels of circRNAs (mm9_circ_004072, mm9_circ_007155, mm9_circ_010239, mm9_circ_013197), miRNAs (miR-21a-3p, miR-205-5p, miR-208-3p, miR-202-5p, miR-215-5p, miR-122-3p) and mRNAs (Aqp1, Acta1, Scp2, Tgfb3, Thoc2) were validated.

A total of 2227 miRNA-mRNA pairs containing 43 miRNAs and 210 target mRNAs were found in the RV tissue. In GO and KEGG analyses of target mRNAs, we found that the mRNAs upregulated in PAC mice were significantly enriched in cell growth processes, including positive regulation of the cell cycle, Ras protein signal transduction and PI3K-Akt signaling. The mRNAs downregulated in the RV of PAC mice were mainly enriched in metabolic processes such as the PPAR signaling pathway.

In renal tissue, 140 miRNA-mRNA pairs were identified. The upregulated mRNAs were enriched in migration and proliferation processes, including positive regulation of cell migration, small GTPase-mediated signal transduction and the PI3K-Akt signaling pathway Moreover, the mRNAs downregulated in the kidneys of PAC mice were also enriched mainly in metabolic processes, including lipid metabolic process, the phospholipase D signaling pathway and the apelin signaling pathway.

Last, we constructed a ceRNA network consisting of RV and kidney circRNAs, miRNAs and mRNAs. The results revealed a consistent finding that under the circumstance of RV dysfunction-induced type II CRS, the most significantly dysregulated pathways in the RV and kidney involved hypertrophy, fibrosis and metabolic alterations.

The ceRNA pairs that were significantly upregulated in the heart were found to be mainly enriched in cardiac hypertrophy-related processes such as the cGMP-PKG pathway. Activation of the cGMP-PKG pathway has been recognized as having a cardioprotective effect [26]. Several miRNAs, such as miR-134-5p, miR-27a-5p, and miR-342-3p, target the PKG downstream molecule Creb1; novel_circ_000915 can also target Creb1 and function as a ceRNA pair according to our prediction. In colon cancer, miR-433 inhibits Creb1 and exerts an antitumor effect by inhibiting the cell cycle [27]. Creb1 is also a direct target of miR-122, which can promote cell proliferation and invasion in bladder cancer [28]. We suspect that a similar mechanism may be involved in the development of RV dysfunction.

Fibrosis is considered a unifying pathophysiology of the CRS continuum [29]. The PI3K-Akt pathway is a classic signaling pathway involved in cardiomyocyte proliferation and fibrosis [30], and a number of circRNAs and miRNAs have reported to participate in the pathophysiological process of CRS. For example, dampening of miR-215 enhances fibroblast cell cycling and proliferation in ocular diseases [31, 32]. MiR-150 appears to modulate sialylation of EGFR via the PI3K/Akt pathway in T-cell acute lymphoblastic leukemia [33, 34], and circRNA_000203 was found to promote cardiac fibrosis by suppressing targets of miR-26b-5p, Col1a2 and CTGF [35]. In this study, we found that the PI3K-Akt pathway was upregulated in both the heart and kidney in PAC mice. Based on the predicted regulatory network, miRNA-215, miRNA-150 and miR-26b-5p are involved in fibrotic- and proliferative-related pathways by targeting mRNAs such as FoxO4 and Kremen1, indicating that miRNA-mRNA pairs and ceRNA interactions play an important role in the fibrotic and proliferative process in our model. MiRNAs also regulate the PI3K/Akt pathway in renal diseases [36], including miR-195, miR-200b, miR-29b and miR-182 [37–39], indicating that after RV failure, hypoxic cardiomyocytes initiate repair and regeneration programs to compensate for the damage caused by hypoxia and fibrosis. In the kidney, low cardiac output causes a decrease in renal blood flow, hypoxia [40] and dysregulation of related noncoding RNAs, subsequently inducing dysregulation of target mRNAs and activation of signaling pathways involved in hypoxia-induced renal fibrosis, including cGMP-PKG and PI3K-Akt pathways [41, 42].

Another important finding in our study is that the mRNAs downregulated are primarily enriched in metabolic processes both in the heart and kidney. In the failing heart, glucose and fatty acid oxidation decreases, and the main metabolic mode switches to glycolysis [43], which is consistent with our findings. According to our results, significantly downregulated miRNA-mRNA pairs are mainly enriched in metabolic processes such as the PPAR signaling pathway. PPAR-α is involved in fatty acid metabolism. It has been reported that miR-21-5p inhibition in type IV CRS can reduce LV hypertrophy and improve LV function by targeting PPAR-α to protect 5/6 nephrectomy rats, with no significant effect on the pathology of the kidney [15]. In our miRNA-mRNA network, Ppargc1a was the most significantly downregulated mRNA, and miR-296-y, miR-199-x and other miRNAs were predicted to be upstream molecules, indicating that these miRNAs may regulate fatty acid metabolism by inhibiting Ppargc1a, potentially alleviating the damage caused by cardiac metabolic switching. Kang HM et al. demonstrated that deletion of fatty acid metabolism in tubular epithelial cells of the kidney resulted in increased intracellular lipid deposition and contributed to renal fibrosis [44]. In our study, lipid metabolic processes, such as the phospholipase D signaling pathway, were also downregulated, suggesting that along with the fibrotic and proliferative changes of the kidney, metabolic alterations are noteworthy in the progression of CRS.

Using the ceRNA network we constructed, we found the thyroid hormone signaling pathway to be significantly downregulated in heart and kidney tissues. The thyroid hormone signaling pathway is divided into classic thyroxine receptor (TR) α and β pathways and nonclassical TR α and β signaling pathways. The classic pathway comprises negative feedback loop regulation of the hypothalamus-pituitary-thyroid axis. In recent years, it has been reported that activation of nonclassical TRβ pathways can increase energy metabolism and body temperature and reduce exercise capacity and triglyceride concentrations in mice [45]. Hdac4 was also observed to be an important molecule in thyroid signaling in our ceRNA network. According to our prediction, novel_circ_005533 can compete with miR-133a-3p and miR-140-5p to regulate Hdac4, which may be one of the metabolic regulation mechanisms in type II CRS.

Through key node screening in the ceRNA network, we observed multiple related nodes for novel_circ_002631, suggesting that it may play an important role in this pathophysiological process. Regardless, research on this circRNA is lacking, and mechanistic studies of this novel circRNA should be carried out.

In summary, we established a type II CRS mouse model induced by RV dysfunction and analyzed expression patterns of circRNAs, miRNAs and mRNAs in the pathophysiological process of this disease. This study provides useful information for understanding the transcriptome changes occurring in type II CRS induced by RV dysfunction.

### Study limitations

Few studies have been carried out to elucidate the molecular mechanisms of RV dysfunction-induced type II CRS. To achieve a comprehensive understanding of potential mechanisms and therapeutic targets in type II CRS, we focused on bioinformatics analysis in this study. Detailed research on several selected targets will be carried out in our future studies. However, there are several limitations of this study that should be acknowledged. First, the addition of a treatment group to improve type II CRS would provide further clues to determine what signaling pathways play more important roles in the pathogenesis of this disease model. To evaluate expression profiling changes due to intervention, it might be helpful to establish a targeted genetic approach for future studies. Second, we only performed a single time-point analysis, but whether dysfunction of the heart or kidney develops with time and the molecular signatures may vary at different time points. Further study at different time points after PAC should be conducted to better clarify the dynamic changes in circRNA-miRNA-mRNA interactions. Third, our results were obtained in a mouse model, and further exploration should be performed to determine whether they can be translated to humans. Accumulating evidence suggests that certain noncoding RNA expression patterns are conserved across species [46], and further human-specific studies are necessary to better understand the molecular mechanisms.

## MATERIALS AND METHODS

Procedures were all performed in accordance with our institution’s guidelines for animal research that confirm to the Guide for the Care and Use of Laboratory Animals (National Institutes of Health Publication, 8^th^ Edition, 2011). Approval for the study was granted by Ethical Committee of Nanfang Hospital, Southern Medical University (Guangzhou, China). Mice were kept in standard housing conditions with a light/dark cycle of 12 hours and free access to food and water. The data that support the findings of this study are available from the corresponding author upon reasonable request.

### PAC model

Male C57BL/6 mice aged 7-8 weeks and weighing 22-25 grams were anesthetized by intraperitoneal injection of a mixture of xylazine (5 mg.kg^−1^) and ketamine (100 mg.kg^−1^), intubated with PE-50 tubing, and ventilated with room air using a mouse mini-ventilator. The respiration rate was set between 100 and 110 times per minute. PAC was performed as described previously [47]. After anesthesia, an incision was made in the skin parallel to the second rib, approximately 10 mm in length, with ophthalmic scissors, ensuring that the incision starts from the sternal angle and ends on the left anterior axillary line. Then, the second intercostal space was identified by counting the ribs from the sternal angle. The pectoralis major and pectoralis minor muscles were separated and cut above the second intercostal space to expose this space. The second intercostal space was bluntly penetrated and opened. The parenchyma and thymus were carefully separated until the pulmonary trunk was visible, and the PA and the ascending aorta were bluntly separated. A 6-0 braided silk suture was placed through the connective tissue between the PA and the ascending aorta, and after confirming that there was no bleeding in the PA and aorta, the PA together with a padding needle was ligated. The padding needle was removed immediately after the filling of the pulmonary conus was observed. After closing the chest and the skin, the skin was disinfected with 75% alcohol.

### Echocardiography

Four weeks after surgery, echocardiography measurements (VEVO 2100, Visual Sonic, Toronto, ON, Canada) of the right heart were performed under anesthetic (1.5–2% isofluorane, 2 L/min oxygen flow rate) with mice intubated as reported elsewhere [48, 49]. Briefly, measurements of the RV outflow velocity were obtained from the parasternal short-axis view at the level of the pulmonary valve during end-diastole. After aligning the RV free wall perpendicularly to the transducer, the RV ejection time (ET), RV free wall thickness and RV internal diastolic diameter (RVIDd) were measured. The tricuspid closure-to-opening time (TCOT), tricuspid annular plane systolic excursion (TAPSE) and tricuspid valve E/A ratio (TV E/A) were measured from the apical four-chamber view. The RV myocardial performance index (or Tei index) was calculated as (TCOT-ET)/ET to evaluate RV function [50]. After data collection, the mice were sacrificed by an overdose of pentobarbital (150 mg/kg, i.p) and their hearts and kidneys were extracted for further analysis.

### Histological examination and plasma creatinine measurement

Hearts and kidneys were fixed in 10% formalin and embedded in paraffin by standard protocols. Staining with hematoxylin and eosin (H&E) was performed to evaluate myocardial hypertrophy and Azan-Masson staining was used to assess the fibrosis area [48, 51]. Plasma creatinine (Cr) levels were assessed using a Mouse Cr ELISA Kit (Huabo Deyi Biotech., Beijing, China). ImageJ software was used for quantification.

### qRT-PCR

Total RNA was extracted from mouse cardiac and kidney tissues with a total RNA isolation system (Omega, Norcross, GA, USA). One microliter of RNA was used to measure the quantity and quality with a NanoDrop ND-1000 (Thermo Fisher Scientific, Waltham, MA, USA). Total RNA was converted to cDNA by using oligo (dT) primers with a PrimeScriptTM RT Master Mix (Takara Bio Inc., Shiga, Japan) or Mir-XTM miRNA First-Strand Synthesis Kit (Takara Bio USA Inc., CA, USA). Expression of genes was determined by Quantitect SYBR green real-time PCR (Takara Bio Inc., Shiga, Japan) using the primers listed in Table 7.

**Table 7:** Sequences of the primers used in the qRT-PCR validation.

**Primer name**	**Primer sequence**
*GAPDH*, F	TGCTGAGTATGTCGTGGAGTCT
*GAPDH*, R	ATGCATTGCTGACAATCTTGAG
*Collagen I a1*, F	CGCTATCCAGCTGACCTTCC
*Collagen I a1,* R	GCCTTCTTGAGGTTGCCAGT
*Collagen III a1*, F	TGCTCCTGTGCTTCCTGATG
*Collagen III a1*, R	GACCTGGTTGTCCTGGAAGG
*NGAL*, F	ATGTCACCTCCATCCTGGTC
*NGAL*, R	CCTGTGCATATTTCCCAGAGT
*Aqp1*, F	AGGCTTCAATTACCCACTGGA
*Aqp1*, R	GTGAGCACCGCTGATGTGA
*Acta1*, F	CCCAAAGCTAACCGGGAGAAG
*Acta1*, R	CCAGAATCCAACACGATGCC
*Scp2*, F	CCTTCTGTCGCTTTGAAATCTCC
*Scp2*, R	GCTTCCTTTGCCATATCAGGAT
*Tgfb3*, F	CCTGGCCCTGCTGAACTTG
*Tgfb3*, R	TTGATGTGGCCGAAGTCCAAC
*Thoc2*, F	CACATAACCGTTGAGCCTCTC
*Thoc2*, R	ACTTGCTTCGGTGCTCTCTTG
miR-21a-3p	CAACAGCAGTCGATGGGCTGTC
miR-205-5p	TCCTTCATTCCACCGGAGTCTG
miR-208-3p	ATAAGACGAACAAAAGGTTTG
miR-202-5p	TTCCTATGCATATACTTCTTT
miR-215-5p	ATGACCTATGATTTGACAGAC
miR-122-3p	AAACGCCATTATCACACTAAAT
mm9_circ_004072, F	GCTTGCTCTTCGTCTTGT
mm9_circ_004072, R	TCGGTCCACTTGATGCTA
mm9_circ_007155, F	GGAGAAGGTGAGAGGAAGA
mm9_circ_007155, R	CAGCAGTGGTGTTGAGAT
mm9_circ_010239, F	AGAAGTCGCAGGAGAAGA
mm9_circ_010239, R	CGAGTGCTGAAGATAGGTT
mm9_circ_013197, F	GGTAATACAACAGTGGATGAG
mm9_circ_013197, R	ATGGTGGAGGTAGATAGCA

F: Forward Primer, R: Reverse Primer.

### RNA purification, library construction and sequencing

After total RNA was extracted from mouse cardiac and renal tissues, RNA molecules in a size range of 18–30 nt were enriched by polyacrylamide gel electrophoresis (PAGE). The 3’ adapters were added, and 36-44 nt RNAs were enriched; 5’ adapters were then ligated to the RNAs. The ligation products were reverse transcribed by PCR amplification, and 140-160 bp PCR products were enriched to generate a cDNA library that was sequenced using Illumina HiSeqTM 2500 by Gene Denovo Biotechnology Co. (Guangzhou, China). Clean reads obtained were searched against the miRBase database (http://www.mirbase.org/. Release 21) to identify known miRNAs.

### Gene Ontology (GO) and Kyoto Encyclopedia of Genes and Genomes (KEGG) analyses

MiRNAs and mRNAs with a fold change of ≥ 2 or ≤ -2 and false discovery rate (FDR)<0.05 were considered significantly differentially expressed, and chosen for further analysis. GO and KEGG pathway analyses were conducted to investigate the functions of all DEmRNAs and DEmiRNAs identified in this study. DERNAs were mapped to GO terms in the Gene Ontology database (http://www.geneontology.org/) and significantly enriched GO terms were used to examine DERNAs from a biological process perspective. KEGG pathway analysis (http://www.genome.jp/kegg/) was performed to reveal pathway clusters related to the DERNAs. FDR was calculated to correct *P* values.

### MiRNA target prediction and sponge analysis

RNAhybrid (v2.1.2) + svm_light (v6.01), Miranda (v3.3a) and TargetScan (Version:7.0) were used to predict miRNA targets. miRNA sequences and family information were obtained from TargetScan website (http://www.targetscan.org/). For circRNAs that have been annotated in circBase, the target relationship with miRNAs can be predicted by StarBase (v2.0). For novel circRNAs, three software programs Mireap, Miranda (v3.3a) and TargetScan (Version:7.0) were used to predict targets in animal samples.

### Construction of the circRNA-miRNA-mRNA network

To identify interactions among DEcircRNAs, DEmiRNAs and DEmRNAs, we constructed a coexpression network based on a correlation analysis of the DERNAs. Correlation of expression between miRNA targets was evaluated using the Pearson correlation coefficient (PCC). Pairs with PCC<-0.7 and *P*<0.05 were selected as coexpressed negative miRNA-mRNA target pairs, and all RNAs were differentially expressed. For prediction of mRNAs interacting with circRNAs and miRNAs, miRTarBase (v6.1) was used to predict mRNAs targeted by miRNA sponges, and the circRNA-miRNA-mRNA coexpression network was visualized using Cytoscape software (v3.6.0) (http://www.cytoscape.org/).

### Statistical analysis

Quantitative data are reported as the mean ± standard error. Statistical analysis for comparison between two groups was performed using two-tailed unpaired t-test. All analyses were performed with GraphPad Prism 7.0 software (GraphPad Software, Inc., CA, USA). A *P* value < 0.05 was deemed statistically significant.

## Supplementary Material

Supplementary Figures
