# Supplementary material for: RNA interactions in right ventricular dysfunction induced type II cardiorenal syndrome
Source: Aging (Albany NY). 2021 Jan 20;13(3):4215–41. doi: 10.18632/aging.202385 (PMC7906202; doi:10.18632/aging.202385)
Supplement: Supplementary Figures [file aging-13-202385-s001.pdf]

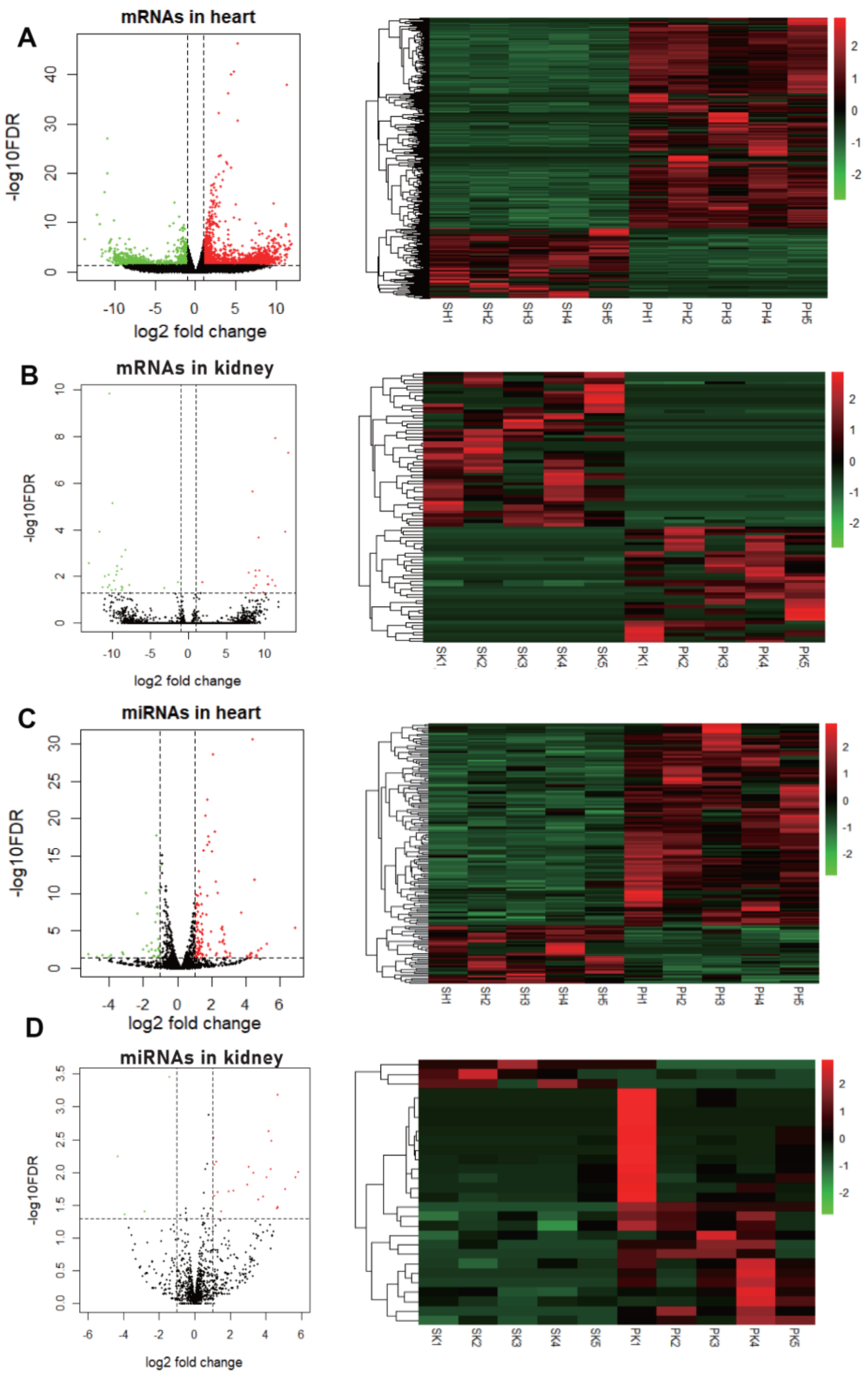

Supplementary Figure 1. Differential expression of mRNAs and miRNAs. (**A**, **B**) Volcano plot and hierarchically clustered heat map illustrating differentially expressed (FDR ≤ 0·05 and the abstract value of $\log_2$(Fold Change) ≥ 1·0) mRNAs of RV and renal tissue samples between sham and PAC group. (**C**, **D**) Volcano plot and hierarchically clustered heat map illustrating differentially expressed miRNAs of RV and renal tissue samples between sham and PAC group. Up-regulated mRNAs and miRNAs are denoted in red and down-regulated ones in green. SH: the RV tissue sample of the sham group; PH: the RV tissue sample of the PAC group; SK: the kidney tissue sample of the sham group; PK: the kidney tissue sample of the PAC group; PAC: pulmonary artery constriction.

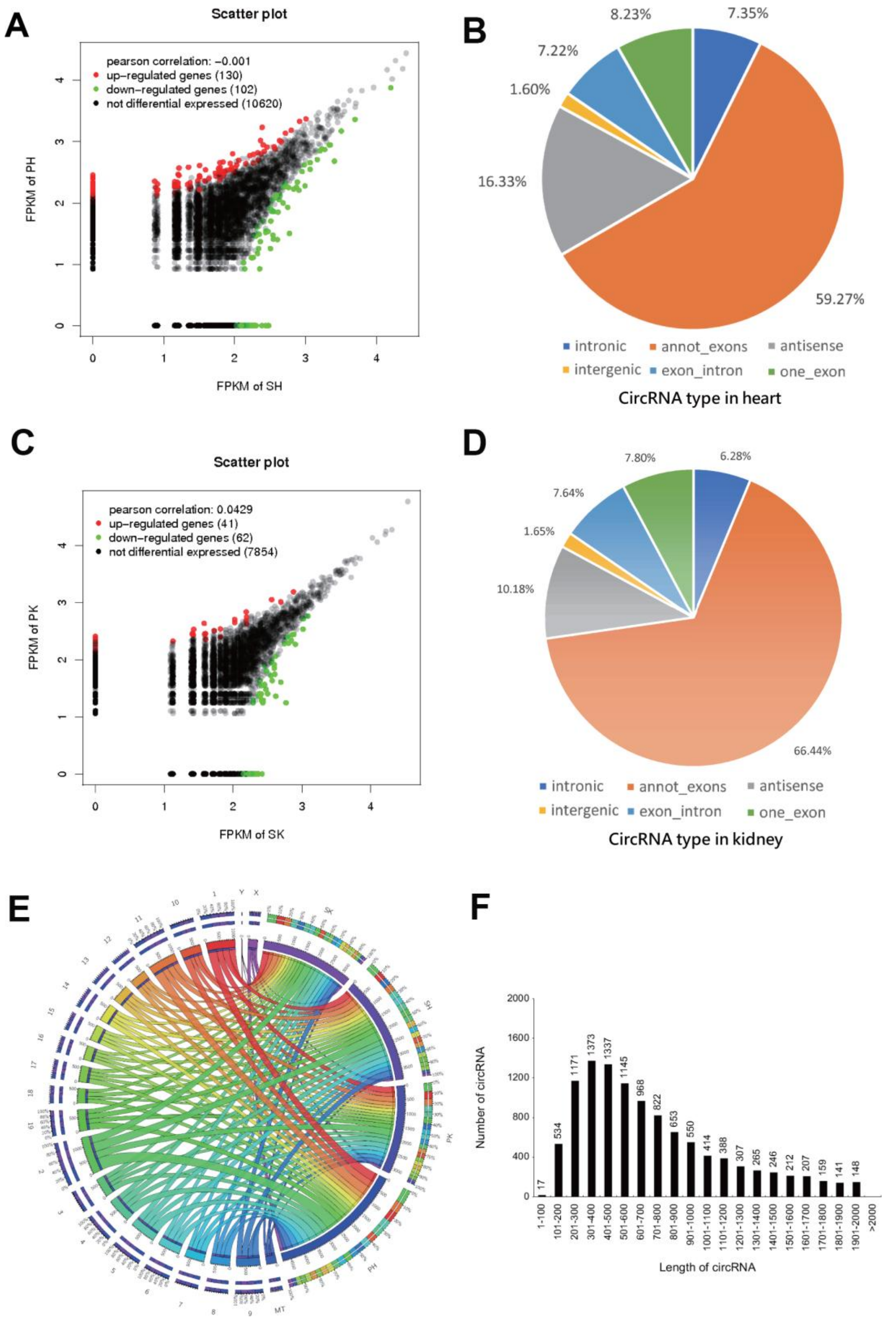

Supplementary Figure 2. Differential expression of circRNAs. (**A**) Scatter plot of the upregulated and downregulated circRNAs in the RV tissue samples between sham and PAC group; (**B**) Subgroups of the DEcircRNAs according to their genomic locations and effect; (**C**) Scatter plot of the upregulated and downregulated circRNAs in the renal tissue samples between sham and PAC group; (**D**) Subgroups of the DEcircRNAs according to their genomic locations and effect; (**E**) Chromosome distribution of the circRNAs; (**F**) The length distribution of circRNAs. SH: the RV tissue sample of the sham group; PH: the RV tissue sample of the PAC group; SK: the kidney tissue sample of the sham group; PK: the kidney tissue sample of the PAC group; PAC: pulmonary artery constriction.

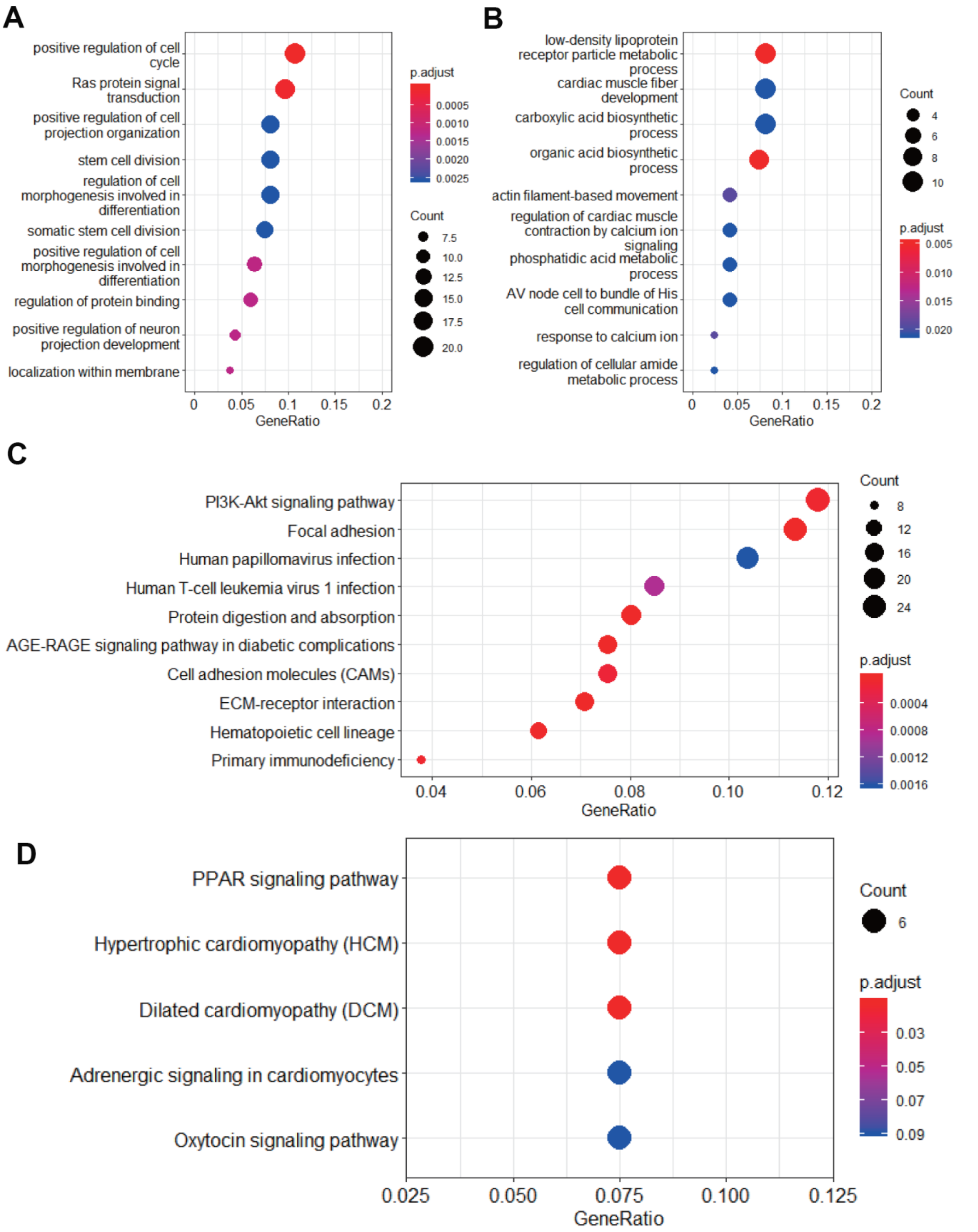

Supplementary Figure 3. Gene Ontology (GO) and Kyoto Encyclopedia of Genes and Genomes (KEGG) analysis of up- and down-regulated mRNAs in RV tissue. (**A**, **B**) GO analysis of the biological functions of differentially expressed mRNAs that were significantly up- and down-regulated in the RV tissue; (**C**, **D**) KEGG pathway analysis of the up- and down-regulated mRNAs in the RV tissue.

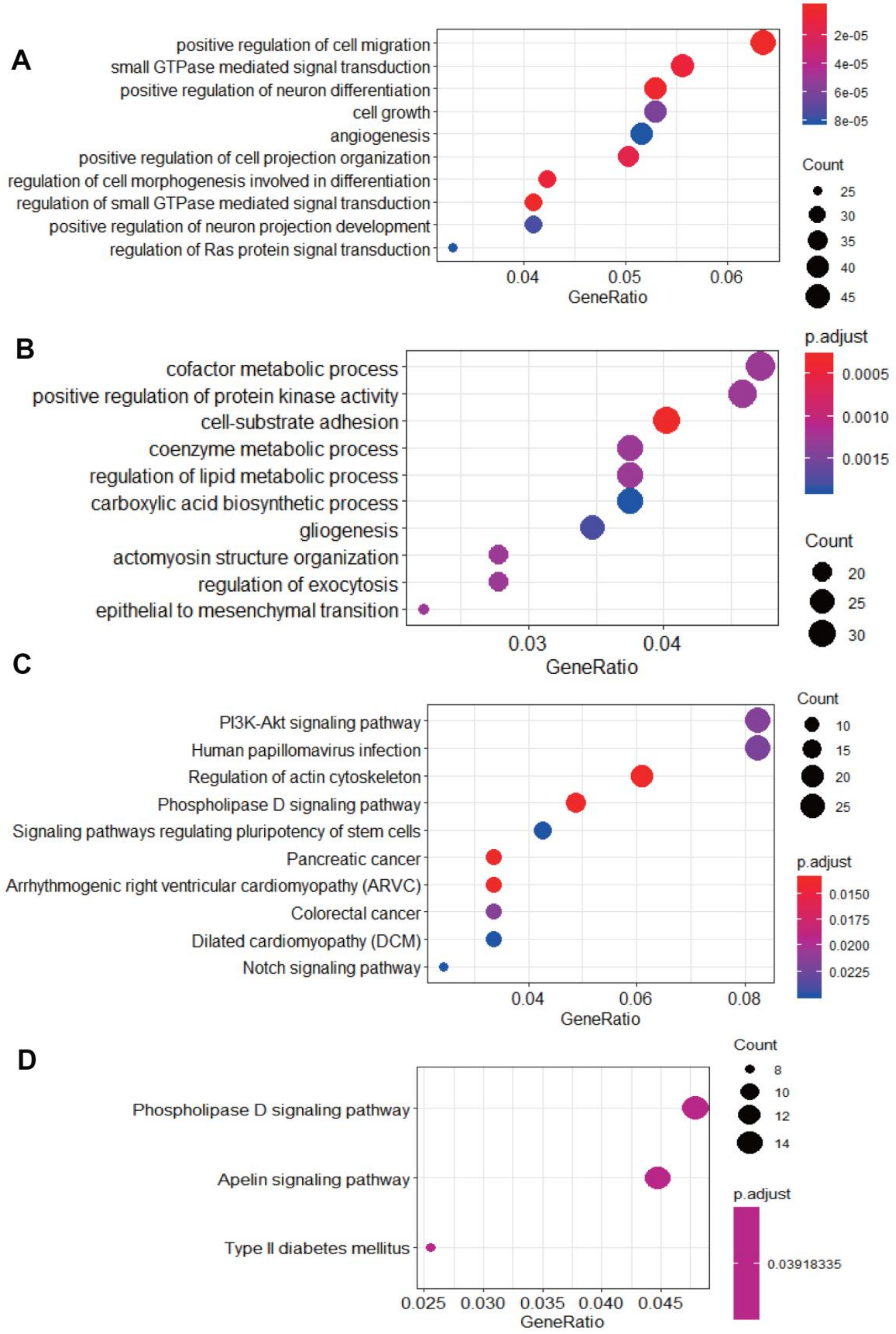

Supplementary Figure 4. GO and KEGG analysis of up- and down-regulated mRNAs in renal tissue. (**A**, **B**) GO analysis of the biological functions of differentially expressed mRNAs that were significantly up- and down-regulated in the renal tissue; (**C**, **D**) KEGG pathway analysis of the up- and down-regulated mRNAs in the renal tissue.

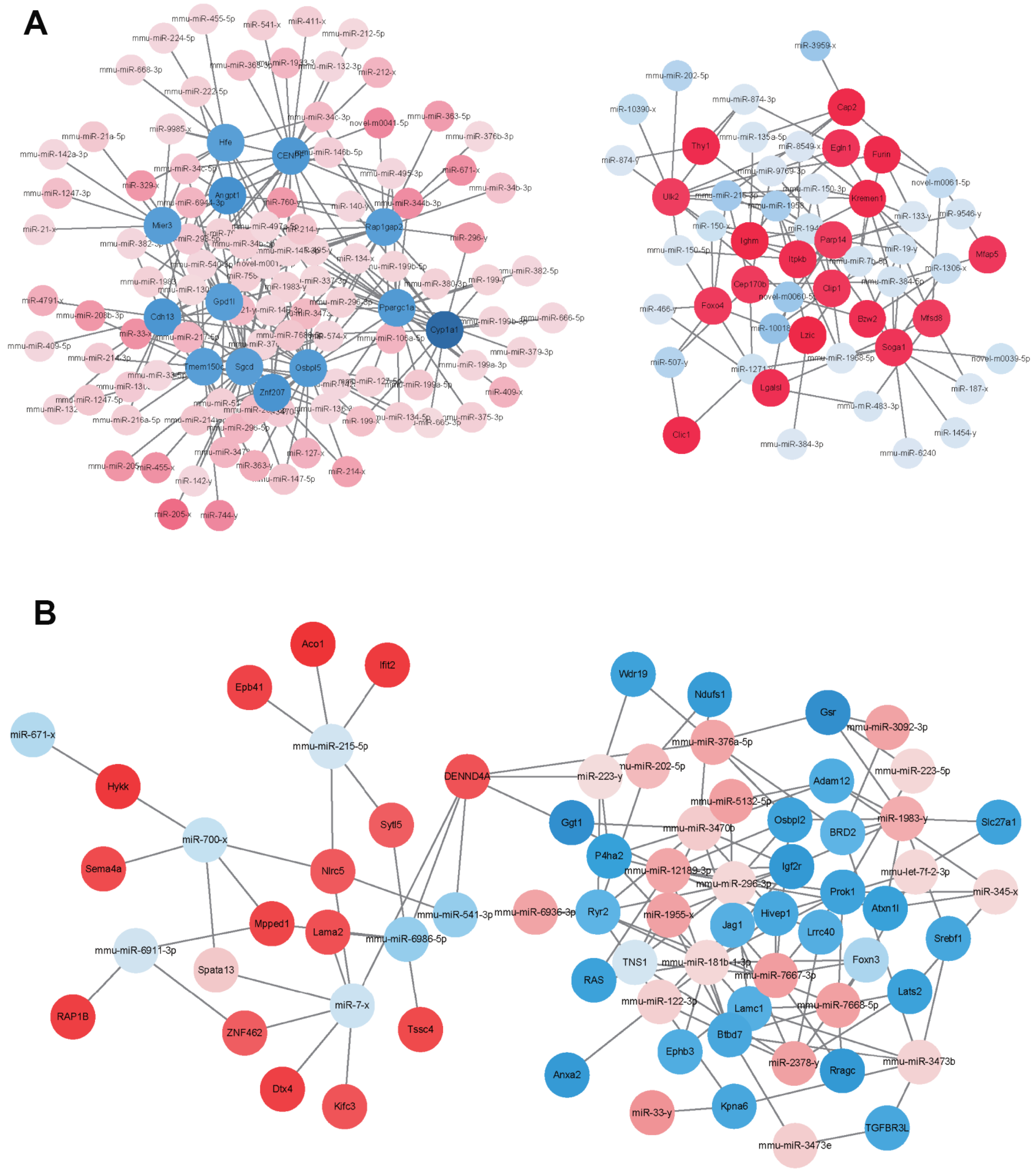

Supplementary Figure 5. Co-expression miRNA-mRNA network. (**A**) The regulatory network of significantly differentially expressed miRNAs (FDR < 0·05 and the abstract value of $\log_2$(Fold Change) ≥ 10·0) and 34 potential target mRNAs; (**B**) The regulatory network of metabolic pathway-related miRNAs and 11 target mRNAs. The red nodes represent up-regulated miRNAs and mRNAs and blue nodes represent down-regulated miRNAs and mRNAs. The $\log_2$(Fold Change) values of the miRNA and target mRNA are shown by the node color. The figures were generated using CytoScape (Shannon et al., 2003).
